# Monckeberg’s Medial Sclerosis in Athletes: Report of Two Cases and Possible Pathophysiological Mechanisms

**DOI:** 10.3390/diagnostics16183064

**Published:** 2026-09-21

**Authors:** Lorenzo Nigris, Peter Pattis, Andrea Lombardi, Bernhard Gutmann, Christian Tröbinger, Federica Ferro, Peter Marschang

**Affiliations:** 1 Department of Internal Medicine, Central Hospital of Bolzano (SABES-ASDAA), Teaching Hospital of Paracelsus Medical University (PMU), 39100 Bozen, Italy; 2 Department of Radiology, Central Hospital of Bolzano (SABES-ASDAA), Teaching Hospital of Paracelsus Medical University (PMU), 39100 Bozen, Italy

**Keywords:** Monckeberg’s medial sclerosis, medial arterial calcification, physical activity, vascular ultrasound, pathophysiological mechanisms

## Abstract

**Background and Clinical Significance:** Monckeberg’s medial sclerosis (MMS) is characterized by the calcification of the tunica media within the arterial wall, resulting in increased arterial stiffness. MMS typically affects the arteries of the lower limbs with elevated ankle–brachial index (ABI) values and preserved peripheral perfusion. Commonly associated conditions are diabetes mellitus and chronic kidney disease (CKD). **Case Presentation:** We report two cases of MMS in middle-aged male individuals without these risk factors, both of whom had a history of long-standing professional athletic activity. In both cases, radiographs demonstrated characteristic “railroad track” medial calcifications, ABI values were elevated, and Doppler ultrasound showed calcifications but preserved triphasic flow without evidence of peripheral artery stenosis or occlusion. **Conclusions:** Given the absence of diabetes, CKD, or smoking history, we hypothesized that repeated strenuous exercise may have triggered the development of arterial calcifications in these two cases. Further research is needed to understand the underlying pathophysiological mechanisms and to determine the clinical and prognostic significance of medial arterial calcifications in athletes.

## 1. Introduction

Monckeberg’s medial sclerosis (MMS) is a condition characterized by the calcification of the tunica media in arterial vessels. Unlike atherosclerotic disease, MMS typically shows preserved perfusion on Doppler ultrasound, although the ankle–brachial index (ABI) may be falsely elevated due to increased vessel wall stiffness [1]. This condition usually involves the lower limb arteries, even though other regions of the body have been described with similar alterations [2]. MMS is regarded as an independent cardiovascular risk factor [3], as well as a risk factor for cerebral infarction [4]. However, most patients with MMS are asymptomatic.

In this article, we describe two cases of MMS that have been recently referred to our outpatient clinic. The patients were not affected by risk factors such as diabetes or chronic kidney disease (CKD), but reported a history of long-lasting professional sports activities. Furthermore, we reviewed the literature and discussed potential pathophysiological pathways for the development of MMS at the molecular level, in murine models as well as in humans.

## 2. Pathophysiology

MMS takes its name from Johann Georg Mönckeberg, who first described this condition in 1903 [5]. MMS is known to affect the medial layer of arterial vessels, as demonstrated by histopathological stains, and is characterized by calcification [6]. By contrast, atherosclerosis primarily affects the intimal layer of arteries.

It is well known that diabetes mellitus, CKD and aging are major risk factors for MMS. Hypertension is known to be a risk factor as well [7,8]. The prevalence of MMS in the diabetic population is particularly high: up to 50% of diabetic patients develop MMS [9]. In terms of arterial stiffness, it is thought that a 40-year-old diabetic patient may have the same level of stiffness as a non-diabetic person at the age of 70 [7]. Males are usually more affected than females. Glycation of molecules, causing the production of advanced glycation end-products (AGEs), may trigger a cascade that activates Runt-related transcription factor 2 (Runx2), a transcription factor that plays a pivotal role in bone formation. The same molecule is then overexpressed in smooth muscle cells, causing calcification of the medial artery wall [7]. When MMS coexists with atherosclerosis, as it often occurs in diabetic patients, MMS can increase the amount of lumen stenosis by accelerating inward remodeling, leading to worsening of clinical symptoms [6]. Moreover, intimal hyperplasia, which may lead to significant lumen loss, is often associated with MMS [7].

In CKD patients, an imbalance of calcium and phosphate metabolism is present. Consequently, the matrix Gla protein (MGP), alkaline phosphatase, osteocalcin and type II collagen are overexpressed. At the same time, inorganic phosphate is increased in muscle cells, which can lead to apoptosis. Subsequently, an osteoblastic-like differentiation of smooth muscle cells occurs, leading to tissue calcification [10]. Patients with CKD are known to have vitamin K deficiency. This deficiency has been linked to aortic and iliac artery calcification [11]. Vitamin K activates several types of proteins of hepatic origin, i.e., coagulation factors, and extra-hepatic origin. Extra-hepatic proteins, such as osteocalcin, Gla-rich protein (GRP) and MGP, appear to play a pivotal role in the calcification of vascular smooth muscle cells. Vitamin K supplementation has been shown to improve arterial stiffness in both healthy individuals and patients with end-stage renal disease (ESRD) [12].

The literature also describes cases of MMS after lumbar sympathectomy for ischaemic limb disease in both diabetic and non-diabetic patients [13]. Further studies are needed to fully understand the mechanisms behind MMS, especially for diabetic patients and, most interestingly, for individuals who lack typical risk factors. There are only a few reports in the literature describing medial sclerosis in individuals without these risk factors [14,15]. The pathophysiology of medial arterial calcification remains unclear, particularly in relation to otherwise healthy individuals. Whereas the most prevalent location for MMS alterations is the lower limb arteries, some reports in the literature describe cases of MMS in uterine vessels [16], temporal [17], lingual and facial arteries [18].

## 3. Role of Genetics and Murine Models

Several murine models have been developed to facilitate a more comprehensive understanding of genetic alterations associated with MMS. The genetic alterations have been classified into three categories: genes related to the metabolism of bone, accessibility of inorganic pyrophosphatase (PPI) and modulation of inorganic phosphate [19]. For instance, osteopontin (OPN) and MGP are proteins implicated in bone formation. It is known that these proteins are also present in sites of ectopic calcifications, such as arteries, where they are normally not present. Keutel syndrome is a rare autosomal recessive disease in humans caused by a defect in the MGP gene resulting in aberrant vascular calcifications.

The development of murine models has facilitated the study of genetic alterations implicated in the process of bone formation. Interestingly, mutant mice negative for both MGP and OPN showed arterial calcifications and other ectopic calcifications. These mice died earlier compared to wild-type mice or mice negative for a single gene (either MGP or OPN) because of vascular rupture, which subsequently resulted in hemorrhage [20].

Imbalances of phosphate metabolism have been observed in other genetic disorders, including generalized arterial calcification of infancy (GACI). In this condition, cells are unable to transform ATP to AMP and phosphate–pyrophosphate (PPi). This condition is usually caused by defects in the gene ectonucleotide pyrophosphatase/phosphodiesterase 1 (ENPP1). Defects in the gene ABCC6 in humans can cause GACI or a condition called pseudoxanthoma elasticum (PXE). PXE manifests during adolescence and adulthood and is usually characterized by a defect of PPi and alterations similar to those of GACI, albeit to a lower extent [19]. A mouse model that is double negative for ENPP1 presents vascular calcifications similar to those observed in humans with GACI. Conversely, ex vivo studies with double-knockout osteoblasts for tissue non-specific alkaline phosphatase and ENPP1 exhibited normal levels of PPi content and normal hydroxyapatite deposition [20].

Several other genes have been identified in both humans and rats to be causing vascular calcifications [21,22,23]. However, the majority of these are monogenic alterations, and the complex pathogenesis of MMS remains to be elucidated [24]. We believe that a variety of genes could explain a predisposition in some patients to develop vascular calcifications.

## 4. Diagnosis

The diagnosis is usually made with clinical presentation, plain radiographs/CT scans and Doppler ultrasound. For example, X-ray images can reveal calcifications of vessels with a typical “railroad track” appearance, especially in the lower limbs (see Cases 1 and 2). Doppler ultrasound typically reveals normal perfusion, with triphasic flow spectra, in the context of medial arterial calcification. ABI is usually increased, i.e., greater than or equal to 1.4, which reflects increased stiffness and non-compressibility of the arterial vessels [1].

## 5. Case Presentation

### 5.1. Case 1

**Figure 1:**
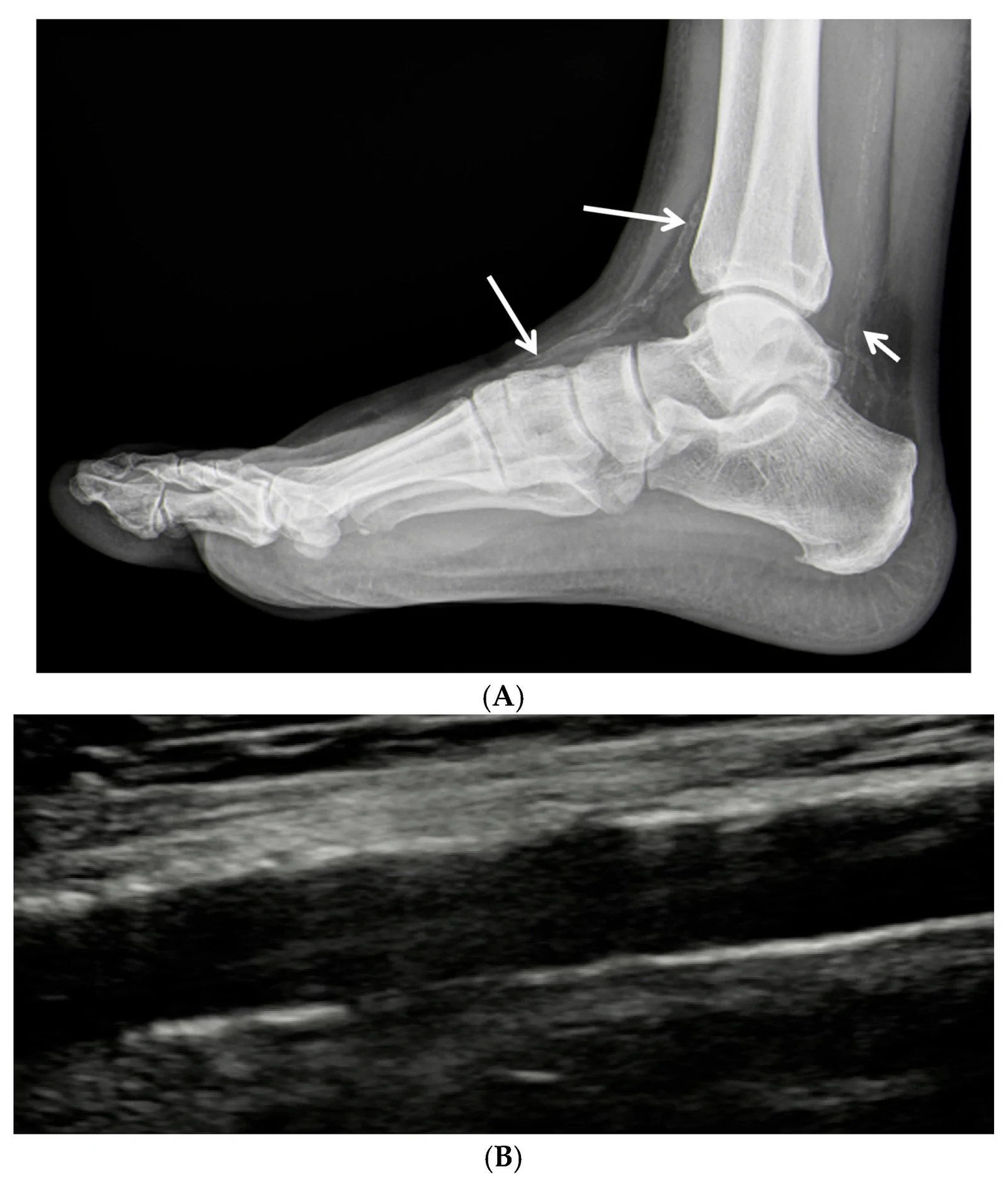
(**A**) X-ray of the right foot with typical “railroad track pattern” calcifications along the anterior and posterior tibial arteries and dorsalis pedis artery (arrows). (**B**) B-mode ultrasound of the left superficial femoral artery with medial calcifications: string-of-beads pattern proximally (left side of the image) transitioning to a confluent calcification distally (right side of the image).

A 52-year-old male patient was referred to our outpatient clinic because of vascular calcifications. These calcifications were discovered incidentally during the course of an X-ray performed for orthopedic indications (see Figure 1A). The calcifications appeared in the form of a ‘railroad track’ pattern in the tibial and foot arteries on both sides. He was asymptomatic regarding his lower limbs. The patient reported being a former professional alpine skier from the age of six until the age of 20. He was treated with a statin for dyslipidemia, but had no previous history of diabetes, hypertension or CKD. The patient had never received any anticoagulation and was a non-smoker.

Peripheral pulses were present in both lower limbs, and the measurement of the ABI indices revealed values of 1.18 on the right side and >1.6, i.e., non-compressible, on the left side.

Doppler ultrasound performed during the visit revealed normal perfusion of the lower limbs. Medial calcifications were identified on both the left and right lower limb arteries, particularly in both superficial femoral arteries (see Figure 1B) and the popliteal arteries. Color-coded duplex ultrasound revealed normal, conserved triphasic flow spectra up to the distal tibial arteries. Doppler sonography of the supra-aortic arteries was also performed, and neither calcifications nor flow alterations were present. Furthermore, a normal intima-media thickness in the common carotid artery (0.6 mm right and 0.7 mm left side) was described.

### 5.2. Case 2

**Figure 2:**
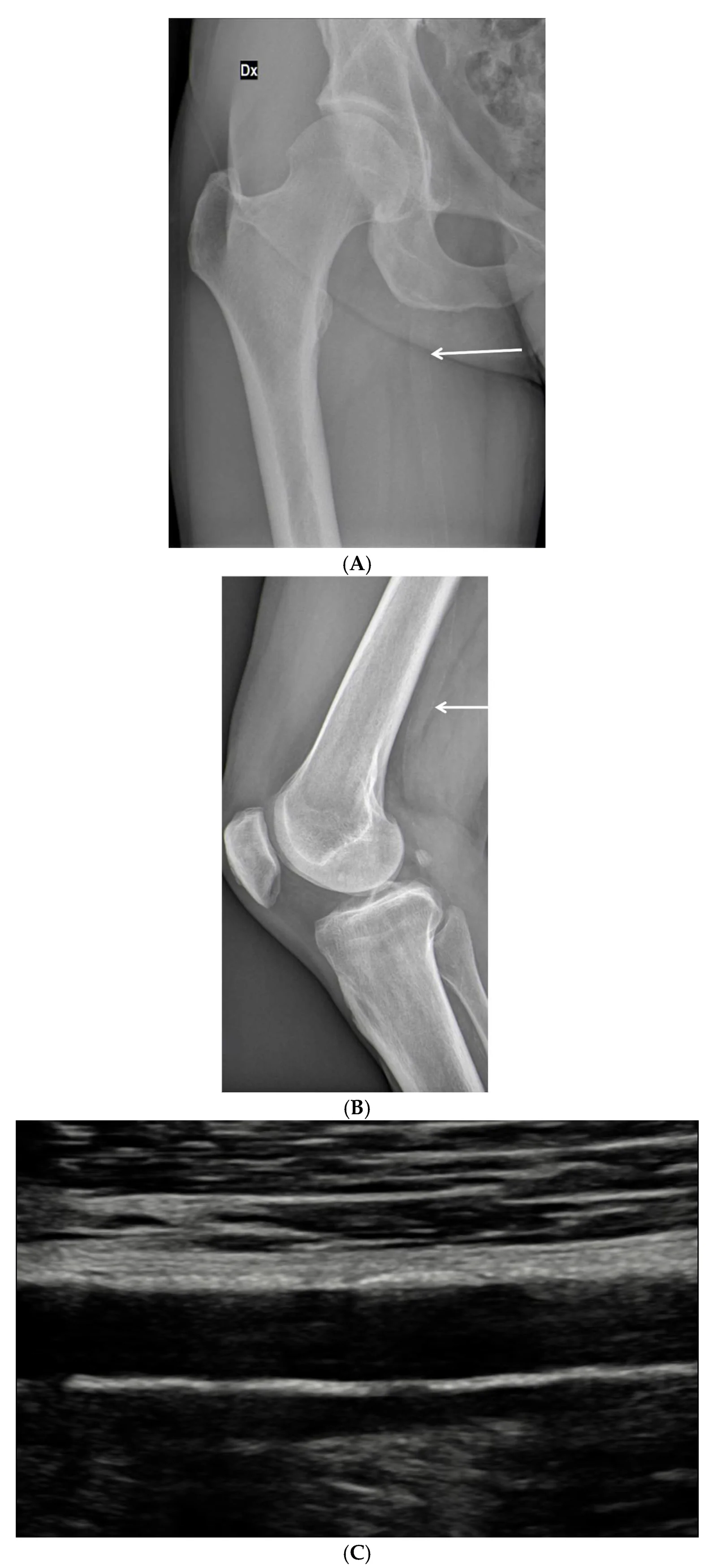

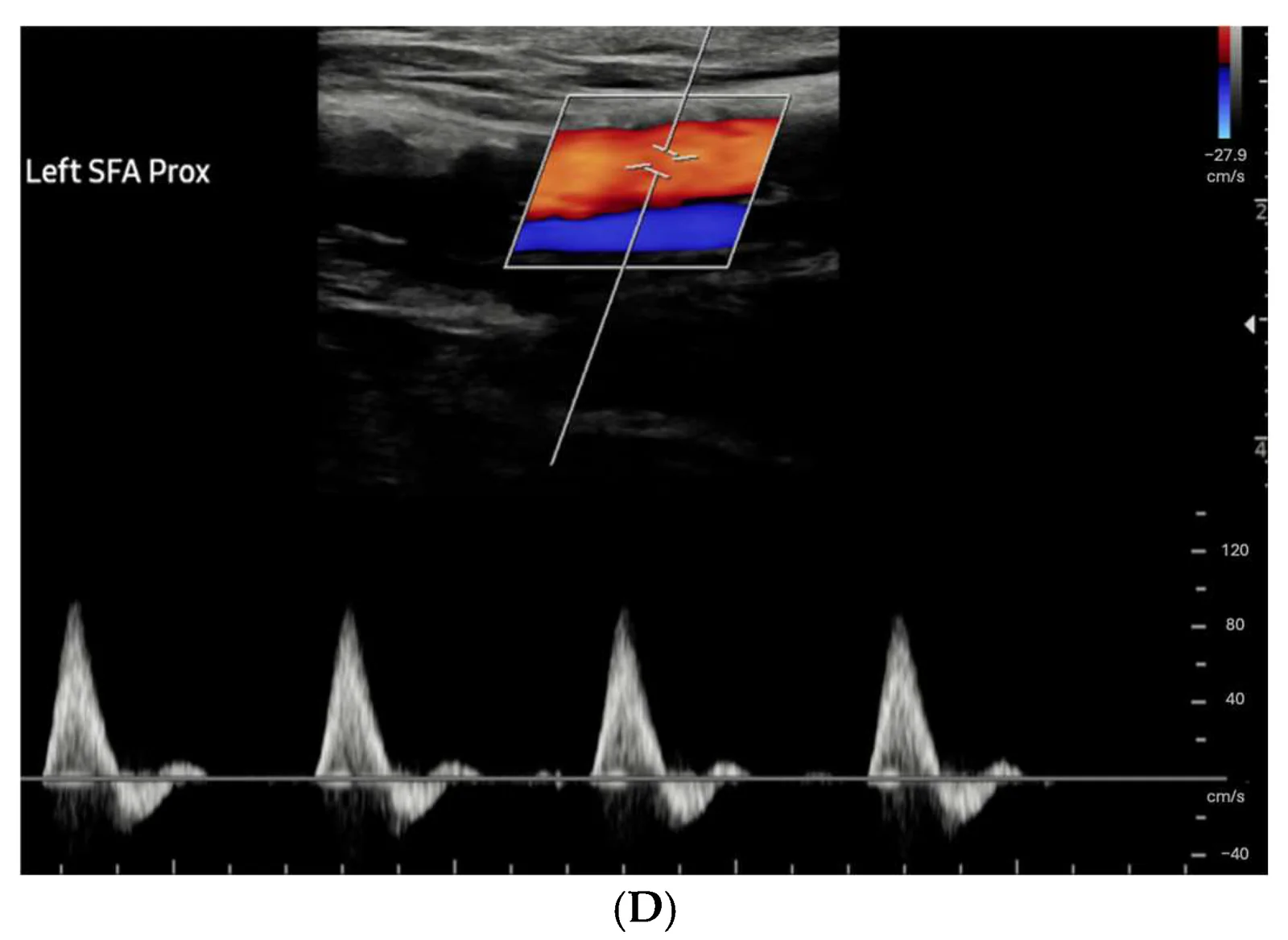
(**A**) Radiography showing typical “railroad track” pattern of the right common femoral artery (arrow). (**B**) Radiography showing typical “railroad track” pattern of the popliteal artery (arrow). (**C**) B-mode ultrasound of the left superficial femoral artery revealing confluent hyperechogenic medial calcification. (**D**) Doppler ultrasound of the left superficial femoral artery, demonstrating normal triphasic flow spectra.

A 58-year-old male patient was referred to our outpatient clinic following an X-ray of his right hip, which revealed diffuse calcification in both the right common femoral and popliteal arteries (see Figure 2A,B). He was asymptomatic as well. The patient reported being a professional basketball player up to the age of 28. He presented with moderately elevated LDL-cholesterol levels (151 mg/dL), normal HDL-cholesterol and normal kidney function. He was a non-smoker and reported no history of diabetes, chronic kidney disease (CKD), or any other cardiovascular risk factors. He was also not taking any medication. A recent echocardiography had revealed no abnormalities. Doppler sonography of the supra-aortic arteries revealed a small, isolated plaque in the proximal left internal carotid artery, without relevant stenosis.

Peripheral pulses were normal bilaterally, and ABI was >1.4 on both limbs. B-mode ultrasound revealed diffuse medial calcifications in the left superficial femoral artery (see Figure 2C). Analogous alterations were also observed in the right superficial femoral artery and bilaterally in the common femoral and popliteal arteries. However, triphasic flow spectra were present on Doppler ultrasound (see Figure 2D), and no signs of occlusive peripheral artery disease were detected.

## 6. Discussion

In this study, we present two cases of MMS in otherwise healthy and asymptomatic male individuals who did not present typical risk factors for MMS such as diabetes or CKD. In both cases, elevated ABI values were measured, and arterial calcifications were documented by X-ray and ultrasound. Importantly, triphasic flow spectra were observed using Doppler ultrasound without signs of significant vessel narrowing or occlusion. Interestingly, both individuals reported a previous history of long-lasting professional sports activity.

In the literature, only a few cases of healthy individuals have been reported to have MMS. A case presentation reported a 46-year-old patient presenting with vascular calcifications who reported regular sports activity, albeit not at a professional level [14].

Another article describes three cases of healthy professional athletes who reported calcifications on lower limb arteries. These patients were runners, while in our cases, the patients were practicing alpine skiing and basketball respectively. In both reports, a similar age range was reported (49–52 years in their study and 52–58 years in our cases) [15].

It is noteworthy that several reports have described calcifications in the coronary arteries of athletes. However, these plaques presented by these individuals are typically calcified, and there is a reduced likelihood of mixed or soft plaques in athletes compared to the normal population. This observation may provide a rationale for the reduced cardiovascular risk observed in athletes, since calcified plaques are less prone to rupture and result in major cardiovascular events [25,26]. Several factors have been cited to explain these alterations, including increased concentrations of parathormone (PTH) [27,28] and inadequate levels of vitamin D [29]. The level of vitamin D has been described as inversely related to coronary artery calcifications [30]. We hypothesize that these hormonal alterations could lead not only to calcifications in coronary arteries, but also in peripheral arteries.

Increased inflammatory responses have been observed during acute exhaustive exercise, while chronic endurance exercise reduces inflammation by activating antioxidant pathways [31]. Although more evidence supports the idea that athletes have lower levels of inflammation, high-intensity athletes may exhibit an acute pro-inflammatory response that, over time, could result in vascular calcifications [32].

Murine models provide evidence that high-intensity training leads to stiffening of the aorta and the carotid arteries as well as medial thickening of the coronary arteries. These vascular changes may be driven by an up-regulation of angiotensin-converting enzyme as well as microRNAs (miRNAs), such as miR-132, miR-212, and miR-146b. Interestingly, mice that underwent moderate training did not exhibit these alterations, suggesting that high-intensity training may be a prerequisite for these pathological alterations [33].

**Figure 3:**
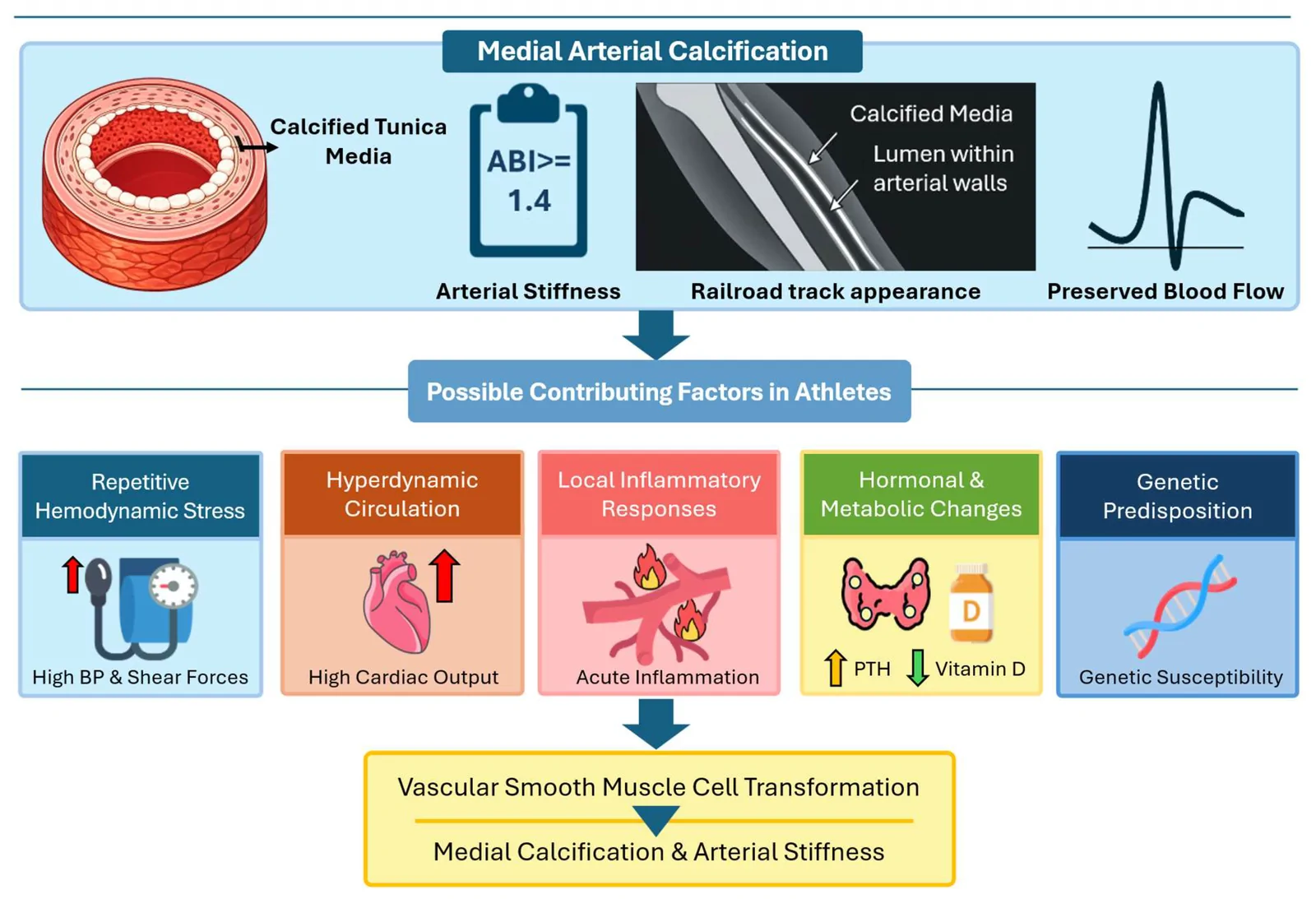
Schematic representation of possible pathophysiological mechanisms of MMS in athletes (Abbreviations: ABI: ankle–brachial index; BP: blood pressure; PTH: parathormone).

**Figure 4:**
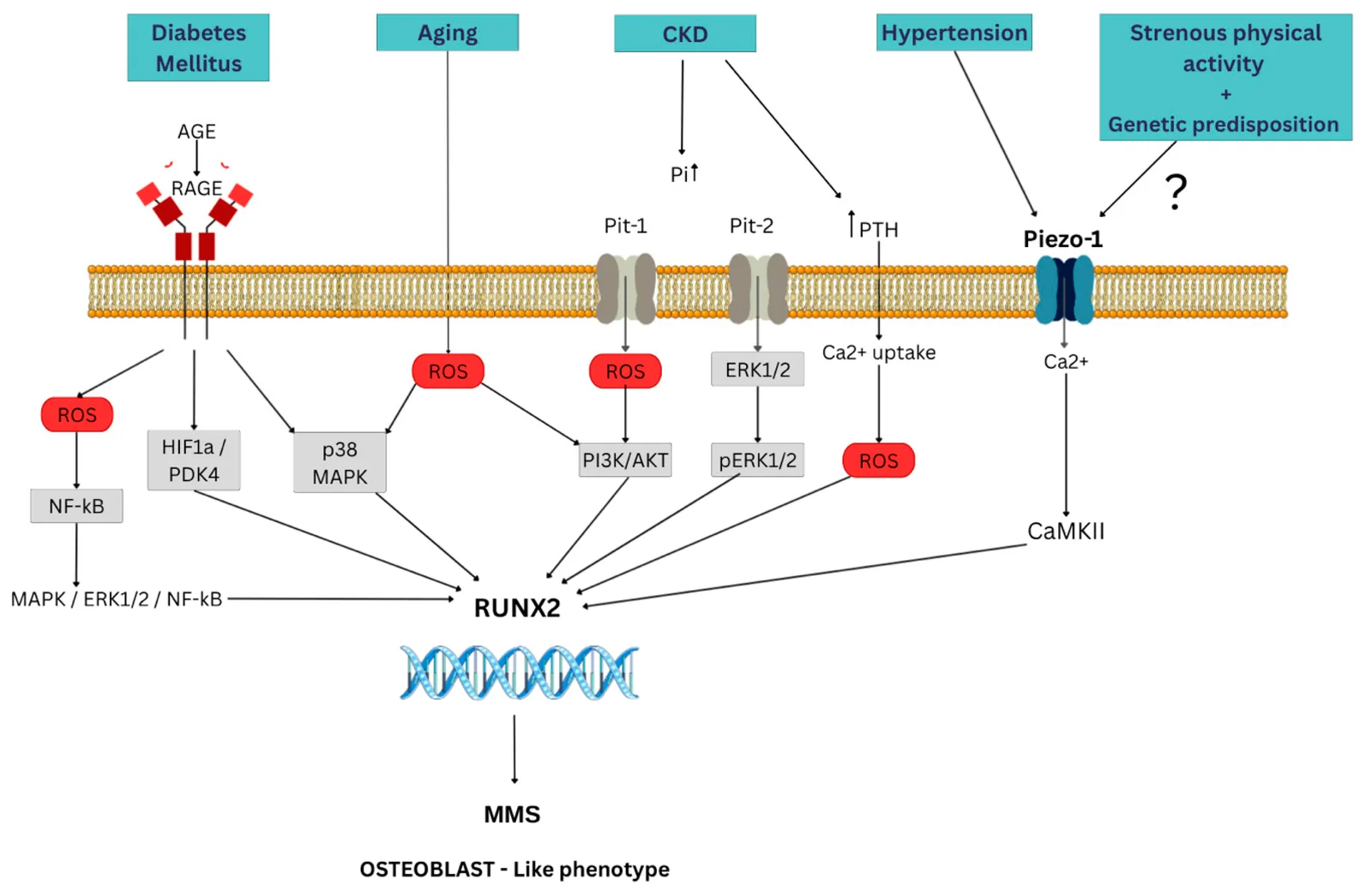
Possible molecular mechanisms involved in MMS (Abbreviations: AGE: advanced glycosylation end-products; CKD chronic kidney disease; CaMKII: calcium/calmodulin- dependent kinase II; ERK 1/2: extracellular signal-regulated kinase 1/2; HIF1α: hypoxia-inducible factor 1α; MAPK: mitogen-activated protein kinase; MMS: Monkeberg’s medial sclerosis; NF-kB: nuclear factor kappa-light-chain-enhancer of activated B cells; PDK4: pyruvate dehydrogenase kinase 4; pERK1/2: phospho-extracellular signal-regulated kinase 1/2; Pi: inorganic phosphate; Pit-1: inorganic phosphate transporter 1; Pit-2: inorganic phosphate transporter 2; PI3K: phosphatidylinositol 3’ kinase; PTH: parathormone; RAGE membrane-bound receptor for advanced glycosylation end-products; ROS: reactive oxygen species; Runx2: Runt-related transcription factor 2).

The molecular mechanisms responsible for the development of medial arterial calcification in different pathologies (aging, diabetes, CKD, hypertension) are not fully understood. In each case, tightly regulated intracellular signaling networks are activated and converge on the activation of the osteogenic transcription factor Runx2 in vascular smooth muscle cells (VSMCs). Once activated, Runx2 drives a canonical osteogenic gene program in VSMCs, ultimately leading to a phenotype switch with the generation of osteoblast-like VSMCs and vascular calcification [7]. In aging, mitochondrial dysfunction and DNA damage, e.g., by telomere shortening, lead to an increased amount of reactive oxygen species (ROS), which activate Runx2 via phosphatidylinositol 3′–kinase (PI3K)/AKT and p38 mitogen-activated protein kinase (MAPK) signaling [32,34]. In diabetes, AGEs interact with the membrane-bound receptor for advanced glycation end-products (RAGE), thereby promoting Runx2-driven osteogenic reprogramming via several intracellular signaling pathways (nuclear factor κB (NF-κB)/MAPK/extracellular signal-regulated kinase 1/2 (ERK 1/2), p38 MAPK, hypoxia-inducible factor 1α/pyruvate dehydrogenase kinase 4 [7]. In CKD, hyperphosphatemia induces the uptake of inorganic phosphate by sodium-dependent phosphate cotransporters Pit-1 and Pit-2, followed by Runx2 activation via PI3K/AKT or ERK 1/2 [7]. In addition, secondary hyperparathyroidism, which is triggered by low vitamin D levels with subsequently elevated extracellular Ca^2+^, activates the uptake of Ca^2+^-containing extracellular vesicles. Increased intracellular Ca^2+^ leads to the activation of NADPH oxidase 5 (Nox5), resulting in increased oxidative stress and contributing to the VSMC phenotype switch [35]. Recent evidence suggests that the mechanosensitive ion channel Piezo1 plays an important role in inducing medial calcification in hypertension. Piezo1 seems to influence calcification by increasing intracellular Ca^2+^ and subsequently activating Runx2 via calcium/calmodulin-dependent protein kinase II (CaMKII) [36,37]. Since Piezo1 has been identified as a sensor of increased shear stress resulting from elevated cardiac output, and more generally as a sensor of physical exercise [38], we propose a similar mechanism in athletes, in whom repeated Piezo1 activation over years of strenuous physical exercise may lead to MMS in the presence of a genetic predisposition (see Figure 3 and Figure 4).

In light of the evidence presented in the two cases and the supporting literature, we hypothesize that a relationship between intense sport activity and medial wall calcification might exist. In patients with a specific genetic predisposition, continuous shear stress forces due to intense sport activities may lead to local stress and inflammation in the tunica media of the leg arteries. Such alterations might be relevant, especially when these stimuli are perpetuated for years on a daily basis.

However, the available evidence supporting this hypothesis is limited. Further research in both human and mouse models is required to provide a better understanding of these pathophysiological alterations and to define a possible prognostic impact of MMS.

## Data Availability

The original contributions presented in this study are included in the article. Further inquiries can be directed to the corresponding author.
